# Recombinant Vesicular Stomatitis Virus Vaccine Vectors Expressing Filovirus Glycoproteins Lack Neurovirulence in Nonhuman Primates

**DOI:** 10.1371/journal.pntd.0001567

**Published:** 2012-03-20

**Authors:** Chad E. Mire, Andrew D. Miller, Angela Carville, Susan V. Westmoreland, Joan B. Geisbert, Keith G. Mansfield, Heinz Feldmann, Lisa E. Hensley, Thomas W. Geisbert

**Affiliations:** 1 Galveston National Laboratory, University of Texas Medical Branch, Galveston, Texas, United States of America; 2 Department of Microbiology and Immunology, University of Texas Medical Branch, Galveston, Texas, United States of America; 3 Harvard Medical School, Boston, Massachusetts, United States of America; 4 Division of Comparative Pathology, New England Primate Research Center, Southborough, Massachusetts, United States of America; 5 Department of Pathology, New England Primate Research Center, Southborough, Massachusetts, United States of America; 6 Division of Primate Resources, New England Primate Research Center, Southborough, Massachusetts, United States of America; 7 Laboratory of Virology, Division of Intramural Research, National Institute of Allergy and Infectious Diseases, National Institutes of Health, Hamilton, Montana, United States of America; 8 Virology Division, U.S. Army Medical Research Institute of Infectious Diseases, Fort Detrick, Maryland, United States of America; Tulane School of Public Health and Tropical Medicine, United States of America

## Abstract

The filoviruses, Marburg virus and Ebola virus, cause severe hemorrhagic fever with high mortality in humans and nonhuman primates. Among the most promising filovirus vaccines under development is a system based on recombinant vesicular stomatitis virus (rVSV) that expresses an individual filovirus glycoprotein (GP) in place of the VSV glycoprotein (G). The main concern with all replication-competent vaccines, including the rVSV filovirus GP vectors, is their safety. To address this concern, we performed a neurovirulence study using 21 cynomolgus macaques where the vaccines were administered intrathalamically. Seven animals received a rVSV vector expressing the *Zaire ebolavirus* (ZEBOV) GP; seven animals received a rVSV vector expressing the *Lake Victoria marburgvirus* (MARV) GP; three animals received rVSV-wild type (wt) vector, and four animals received vehicle control. Two of three animals given rVSV-wt showed severe neurological symptoms whereas animals receiving vehicle control, rVSV-ZEBOV-GP, or rVSV-MARV-GP did not develop these symptoms. Histological analysis revealed major lesions in neural tissues of all three rVSV-wt animals; however, no significant lesions were observed in any animals from the filovirus vaccine or vehicle control groups. These data strongly suggest that rVSV filovirus GP vaccine vectors lack the neurovirulence properties associated with the rVSV-wt parent vector and support their further development as a vaccine platform for human use.

## Author Summary

Ebola and Marburg viruses are categorized as Category A priority pathogens by several US Government agencies as a result of their high mortality rates and potential for use as agents of bioterrorism. There are currently no vaccines or therapeutics approved for human use. A replication-competent, recombinant vesicular stomatitis virus (rVSV) vector expressing filovirus glycoproteins (GP), in place of the VSV G protein has shown promise in lethal nonhuman primate models of filovirus infection as both a single-injection preventive vaccine and a postexposure treatment. Replication-competent vaccines that are intended for use in humans usually undergo neurovirulence testing as was done for measles virus, mumps virus, yellow fever virus, and poliovirus vaccines. Here we used a conventional neurovirulence test to evaluate the safety of our rVSV-based *Zaire ebolavirus* and *Lake Victoria marburgvirus* GP vaccines in cynomolgus macaques. Importantly, we demonstrate for the first time that these rVSV filovirus GP vectors lack neurovirulence when compared to a rVSV wild-type vector.

## Introduction

The order *Mononegavirales* comprises the non-segmented, negative sense, single-stranded RNA viruses in the family *Filoviridae* along with the families *Rhabdoviridae*, *Paramyxoviridae*, and *Bornaviridae*. The family *Filoviridae* contains two genera, *Ebolavirus* (EBOV) and *Marburgvirus* (MARV) [1], [2]. Infection with EBOV or MARV causes severe and often fatal hemorrhagic fever (HF) with case fatality rates ranging from 23–90% depending on the strain and/or species. The *Ebolavirus* genus is diverse and consists of four species: *Sudan ebolavirus* (SEBOV), *Zaire ebolavirus* (ZEBOV), *Cote d'Ivoire ebolavirus* (CIEBOV), and *Reston ebolavirus* (REBOV). A putative fifth species, *Bundibugyo ebolavirus* (BEBOV) was discovered during an outbreak in Uganda during 2007 [3]. Together, the *Ebolavirus* genus has accounted for at least 22 outbreaks dating back to 1976 with18 of these occurring within the last 20 years [4]. The *Marburgvirus* genus has one species, *Lake Victoria marburgvirus*, that has been responsible for at least nine outbreaks since 1967 with five of these occurring in the last decade [4]. The increased frequency of EBOV and MARV outbreaks along with the fact that these viruses are potential agents of bioterrorism has increased public health concern regarding filoviruses. Presently, there are no licensed vaccines or postexposure treatments available for human use; however, there are at least six different vaccine candidates that have shown the potential to protect nonhuman primates (NHP) from lethal EBOV and/or MARV infection [5], [6], [7], [8], [9], [10], [11], [12], [13], [14], [15], [16], [17], [18], [19], [20].

The filoviruses contain an RNA genome, approximately 19 Kb in length, which encodes seven proteins that are arranged from 3′ to 5′: nucleoprotein (NP); virion protein (VP)35; VP40; glycoprotein (GP); VP30; VP24; and the polymerase protein (L). The EBOV species express two extra nonstructural proteins from the GP gene referred to as soluble (s)GP [2] and small soluble (ss)GP [21]. Vaccine studies that have shown protection in animals from filovirus infections have primarily employed the GP protein as the immunogen with a few studies also using VP40 and/or NP [5], [6], [7], [8], [9], [10], [11], [12], [13], [14], [15], [16], [17], [18], [19], [20].

*Vesicular stomatitis virus* (VSV), a member of the family *Rhabdoviridae*, is composed of a genome of approximately 11 Kb which encodes five proteins that are arranged from 3′ to 5′: nucleoprotein (N), phosphoprotein (P), matrix protein (M), glycoprotein (G), and the large catalytic subunit (L) of the RNA-dependent RNA polymerase. Over the last decade the use of recombinant (r)VSVs as vaccine candidates has been studied due to the ability to insert and express foreign genes in the simple genome, the ability to propagate the virus to high titers in most mammalian cell lines, the lack of recombination or insertion into the host cell genome, the extremely low percentage of VSV seropositivity in the general population of Central America [22], [23], and the minimal pathogenicity of VSV in humans. rVSV vectors have been developed as vaccine candidates against many important human pathogens such as influenza virus [24], human immunodeficiency virus (HIV) [25], [26], [27], measles virus [28], [29], respiratory syncytial virus [30], papillomavirus [31], [32], severe acute respiratory syndrome coronavirus [33], and HF viruses such as Lassa, EBOV, and MARV [34].

The rVSV filovirus GP vaccine platform, where the VSV glycoprotein (G) is replaced with filovirus GP, has shown promise as both a single-injection preventive vaccine [8], [9], [11], [12], [18], [35] and a postexposure treatment against EBOV and/or MARV challenge in NHPs [36], . Initial studies showed that a single intramuscular (i.m.) vaccination of cynomolgus macaques with a rVSV vector expressing the ZEBOV GP or MARV GP induced strong humoral and/or cellular immune responses and elicited complete protection against a high dose (1000 plaque forming unit [PFU]) i.m. challenge of homologous ZEBOV or MARV given 28 days later [8]. The efficacy of these vaccines to protect by i.m. vaccination against homologous high dose (1000 PFU) aerosol challenge was also tested. Importantly, homologous aerosol challenge with either ZEBOV or MARV 28 days after vaccination resulted in complete protection of the macaques [11]. In addition to vaccinating via the i.m. route, the ability of the rVSV-ZEBOV-GP vaccine to protect cynomolgus macaques by intranasal and oral routes was also studied [18]. Vaccination by these different routes resulted in complete protection against homologous challenge and elicited robust ZEBOV GP-specific humoral responses and T-cell responses that were induced after vaccination and also 6 months after ZEBOV challenge [18]. More recently, we showed that a single injection of a blended vaccine consisting of equal parts of rVSV-ZEBOV-GP, rVSV-SEBOV-GP, and rVSV-MARV-GP completely protected NHPs against lethal challenge with either ZEBOV, SEBOV, CIEBOV, or MARV [12].

While the rVSV filovirus GP vectors have proven robust as preventive vaccines, these vectors have also shown utility as postexposure treatments with the rVSV-MARV-GP vector protecting 100%, 83%, and 33% of rhesus macaques challenged with MARV and treated 30 minutes, 24 hours, and 48 hours postexposure, respectively [36], [39]. Treatment of macaques 30 minutes after challenge with a rVSV-SEBOV GP vector protected all animals against a lethal SEBOV challenge [38] while treatment of macaques 30 minutes after challenge with the rVSV-ZEBOV GP vector protected 50% of the animals against a homologous ZEBOV challenge [37].

To date, the rVSV filovirus GP vectors have been used in over 80 NHPs with no signs of toxicity as a result of vaccination [8], [9], [11], [12], [18], [35]. In addition, the rVSV-ZEBOV-GP vector was recently used as a treatment less than 48 hours after a possible, accidental ZEBOV exposure to a laboratory worker in Germany. While the efficacy of the treatment was not conclusive, the treated individual experienced mild fever, myalgia, and headache 12 hours after injection [40]. Although these data suggest that the vectors are innocuous, the use of the rVSV filovirus GP vectors as vaccines and/or postexposure treatments in humans requires further safety testing. This is important considering that the vaccine is replication-competent and there is potential for VSV neurovirulence (NV), as has been reported in rodents [41], [42], [43], [44], [45], [46], [47], [48], macaques [49], cattle, sheep, and horses [50]. Regardless of the potential for rVSV vector NV, replication-competent vaccines that are to be used in humans are generally subjected to NV testing. Typically, NV is evaluated by direct inoculation of the vaccine into the central nervous system (CNS) of a NHP. These NV tests have been developed for measles virus [51], [52], mumps virus [53], [54], yellow fever virus [55], [56], and poliovirus [57], [58]. Most recently rVSV HIV vaccine variants have been tested for NV by intrathalamic (IT) inoculation with promising results [59].

In the current study, we evaluated the NV potential of the rVSV-ZEBOV-GP and the rVSV-MARV-GP vectors in cynomolgus macaques. The NV tests were modeled on previous examinations of the yellow fever virus vaccine [55] and rVSV HIV vector variants [59] where cynomolgus macaques were given an IT inoculation with 10^7^ PFU of the vaccine and closely monitored over the course of 21 days.

## Materials and Methods

### rVSV vectors

**Figure 1:**
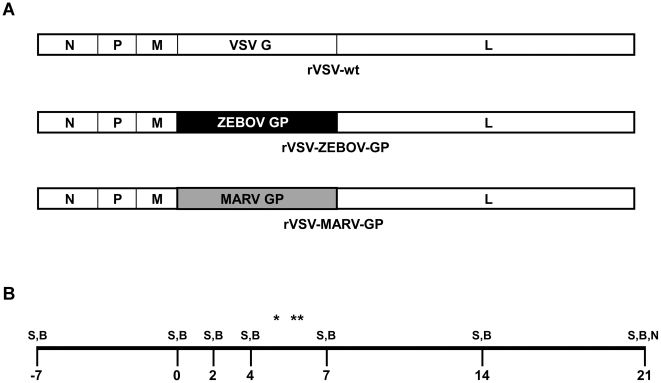
Neurovirulence assay design. (A) Illustration of the rVSV genomes used in the IT inoculation procedures. Note that the only difference between the rVSV vectors used in this study were the glycoproteins. (B) Schematic of the sampling days during the 21 day study. S = swab, B = blood, N = necropsy, * day 5 necropsy of 67-01, ** day 6 necropsy of 56-09.

The rVSV-wt (Fig. 1A) used in this study was recovered from cDNA using methods previously described [60]. The rVSV filovirus GP vectors, rVSV-ZEBOV-GP (Fig. 1A) and rVSV-MARV-GP (Fig. 1A) were recovered from cDNA as previously described [34]. All viruses were propagated by infecting Vero cells at a multiplicity of infection of 0.01 and collecting supernatants when cells showed cytopathic effects. Media used for propagating the rVSVs consisted of Dulbecco's Modified Essential Media, 10% heat-inactivated certified FBS (Invitrogen, Carlsbad, CA), 1× penicillin/streptomycin (Invitrogen), and 1× Glutamax (Invitrogen) (D-10). Virus supernatants or mock-infected cell supernatants (vehicle control) were clarified of cell debris and aliquoted for use in the study and titers determined on Vero cell monolayers by conventional plaque assay.

The rVSV preparations were assessed for the presence of endotoxin using The Endosafe®-Portable Test System (PTS) (Charles River, Wilmington, MA). Virus preparations were diluted 1∶100 in Limulus Amebocyte Lysate (LAL) Reagent Water (LRW) per manufacturer's directions and endotoxin levels were tested in LAL Endosafe®-PTS cartridges as directed by the manufacturer. Each virus preparation was found to be below detectable limits while positive controls showed that the tests were valid.

### Animals

A total of 21 healthy male cynomolgus macaques (*Macaca fascicularis*) (4–7 Kg) were purchased from Charles River Laboratories (Wilmington, MA). All animals ranged in age from 4 to 6 years with the exception of two animals (67-01, 68-01) which were 18 years in age. The study was conducted at the New England Primate Research Center (NEPRC), Harvard Medical School and the animals were given care in accordance with standards of the Association for Assessment and Accreditation of Laboratory Animal Care and the Harvard Medical School Animal Care and Use Committee. All animal work adhered to the regulations outlined in the USDA Animal Welfare Act (9 CFR, Parts 1, 2, and 3) and the conditions specified in the Guide for the Care and Use of Laboratory Animals (ILAR publication, 1996, National Academy Press) and by the Harvard Medical School Animal Care and Use Committee. These experiments and procedures were approved by the Harvard Medical Area Standing Committee on Animals. Any clinical signs of illness or distress were promptly reported to the responsible veterinarian, who recommended treatment for minor ailments or euthanasia when clinical observations and neurological scores of animals reached levels based on the approved Harvard Medical School Animal Care and Use Committee protocol.

### Inoculation procedures

Animals were sedated with Ketamine HCl administered i.m., intubated, and placed on O_2_/Isoflurane. A saphenous catheter was placed and NaCl infused at 10 ml/hr. Atropine, Buprenex, and Cephazolin were administered i.m. The animals head was shaved and surgically prepared. A midline incision was made along the scalp and the skin and musculature was retracted. Two 0.16 cm holes (one per side) corresponding to the positioned incisions on the left and right sides were made through the skull using a high speed general twist drill. Sterile saline was used to flush and clear bony debris during drilling. The inoculum was administered using a 25 gauge, 1.5 inch needle into the thalamic region. Animals received one of four possible inoculates: the three primary inoculates consisted of 1×10^7^ PFU of either rVSV-wt (n = 3), rVSV-ZEBOV-GP (n = 7), or rVSV-MARV-GP (n = 7) in 150 µl of 10% heat-inactivated fetal bovine serum (FBS) in Dulbecco's modified Eagle media. The vehicle control (n = 4) consisted of clarified Vero cell culture supernatant that was 10% heat-inactivated FBS in Dulbecco's modified Eagle media. A small piece of gel foam was placed in the burr hole and direct pressure was used to achieve hemostasis. The musculature and skin were closed in a simple interrupted pattern. Cephazolin was administered for 5 days and Buprenex was administered for 48 hours post procedure (or longer if necessary). The animals were monitored daily by both the veterinary and animal care staff. Each animal was given a thorough physical examination at the time of scheduled phlebotomy. Blood from phlebotomies was monitored for hematology from whole blood and clinical chemistry from serum. Daily cage side neurological exams were conducted. Each animal received an overall impairment score (0–4) and were further characterized neurologically based on specific observation points listed: 0 = No clinical signs of encephalitis; 1 = Rough coat, not eating; 2 = High pitch vocalization, inactive, slow moving; 3 = Shaky movements, tremors, incoordination, limb weakness; 4 = Inability to stand, limb paralysis, moribund.

### Hematology and serum biochemistry

Total white blood cell counts, white blood cell differentials, red blood cell counts, platelet counts, hematocrit values, mean cell volume, mean corpuscular volume, and mean corpuscular hemoglobin concentration were determined from blood samples collected in tubes containing EDTA, using a laser-based hematologic analyzer (Hemavet, Drew Scientific, Waterbury, CT). Concentrations of albumin, amylase, globulin, alanine aminotransferase, aspartate aminotransferase, alkaline phosphatase, gamma-glutamyltransferase, lactate dehydrogenase, glucose, cholesterol, total protein, total bilirubin, direct bilirubin, urea nitrogen, creatinine, creatinine kinase, triglyceride, bicarbonate, calcium, phosphorus, chloride, potassium, and sodium were measured by IDEXX VetConnect (Westbrook, ME) using an Olympus AU5421 biochemistry analyzer (Olympus Americas, Center Valley, PA).

### Necropsy and tissue collection

Animals were euthanized if neurologic signs developed or at the scheduled study end-point, 21 days post inoculation, with an overdose of intravenous sodium pentobarbital and necropsied immediately afterward. Tissues from five brain regions (frontal cortex (FC), occipital cortex (OC), cerebellum (CB), thalamus (TH), and basal ganglia (BG) and three spinal cord (SC) regions (cervical, thoracic, and lumbar) were collected in 10% neutral-buffered formalin. Following one week fixation, the brain was hemisectioned into right and left hemispheres and specified brain regions were trimmed and embedded in paraffin, sectioned at 5 µm, and stained using hematoxylin and eosin (H&E).

### Histopathologic analysis and scoring

H&E stained sections of brain and spinal cord were analyzed for histopathologic changes associated with NV. For each animal, sections of FC, BG, OC, TH, CB, brain stem (BS), and SC were analyzed. Histopathologic changes consistent with NV were double-blind scored based on presence and severity of perivascular infiltrates, gliosis, neurodegeneration, satellitosis, and necrosis. Cumulative assessment of all changes was scored on a severity scale of 0–4 based on previously published work [59]: 0 = no lesions; 1 = minimal perivascular inflammatory infiltrate, no gliosis, no neurodegeneration, no satellitosis, and no necrosis; 2 = mild perivascular inflammatory infiltrate, mild gliosis, no neurodegeneration, no satellitosis, and no necrosis; 3 = moderate inflammatory infiltrate with moderate gliosis, mild neurodegeneration, mild satellitosis, and mild necrosis; and 4 = severe inflammatory infiltrate with moderate to severe gliosis, neurodegeneration, satellitosis, and necrosis. Scores for brain regions were derived for both right and left hemispheres and also combined for total NV. Scores for SC sections were collectively represented as a single number. Statistics were calculated for histology scores using GraphPad Prism 5 software by using an unpaired, two-tailed, t test.

### rVSV TaqMan PCR assay

To assess the viral loads in nasal, oral, and rectal swabs (day 0, 2, 4, 7, 14, and 21), blood (day 0, 2, 4, 7, 14, and 21), tissues (spleen, liver, kidney, heart, axillary lymph node, and adrenal gland), and neural tissues (FC, BG, OC, TH, CB, BS, and SC) RNA was extracted from swabs (Virus RNA kit, Qiagen, Valencia, CA) and tissue sections (RNeasy kit, Qiagen) and TaqMan PCR for the rVSV genome was performed. The rVSV TaqMan assay for detecting VSV genomes and antigenomes was developed using primers and a probe that target the intergenic region (between M and G on the VSV genome) and the M gene as previously described [61]. The rVSV M probe primer (Applied Biosystems, Carlsbad, CA) had the sequence 6-carboxyfluorescein (6FAM)-5′ TTGGCCTGATTGTCG 3′-6 carboxytetramethylrhodamine (TAMRA). One-step real-time PCR (RT-PCR) was done using OneStep RT-PCR kits (Qiagen). All RT-PCR mixtures contained 5 µl of RNA eluate and master mixes were set up following the manufacturer's protocols. Standards and test samples were assayed in triplicate using the CFX96 detection system (Bio-Rad Laboratories, Hercules, CA) with the following cycle conditions: 50°C for 10 min, 95°C for 5 min, and 40 cycles of 95°C for 10 s and 60°C for 30 s. Threshold cycle (*CT*) values representing rVSV genomes and antigenomes were analyzed with CFX Manager Software, and data are shown as log_10_ genome equivalents (GEq). To create the GEq standard, RNA from rVSV-wt stocks was extracted and the number of rVSV genomes was calculated using Avogadro's number and the molecular weight of the rVSV genome [61].

### rVSV isolation

To assess if there was replicating virus in nasal, oral, and rectal swabs (day 0, 2, 4, 7, 14, and 21), blood (day 0, 2, 4, 7, 14, and 21), non-neural tissues (spleen, liver, kidney, heart, axillary lymph node, and adrenal gland), and neural tissues (FC, BG, OC, TH, CB, BS, and SC) virus titers were determined by conventional plaque assay on Vero cell monolayers. Swabs were collected and placed in D-10 media, while tissues were placed in D-10 media and then homogenized before plaque assay.

## Results

### Clinical observations

In order to evaluate the NV potential of the rVSV filovirus GP vectors cynomolgus macaques were inoculated by IT inoculation with 1×10^7^ PFU of either rVSV-ZEBOV-GP (n = 7, Fig. 1A), rVSV-MARV-GP (n = 7, Fig. 1A), rVSV-wt (n = 3, Fig. 1A), or vehicle control (n = 4). Each animal received two inoculations, one per hemisphere, with the left hemisphere receiving the experimental inoculation and the right hemisphere receiving the vehicle control. Animals were then observed for neurologic signs of disease. Clinically, two macaques from the rVSV-wt control group (67-01 and 56-09) developed progressive neurological signs including ataxia, proprioceptive deficits, and tremors, and were euthanized at either day 5 or 6 post inoculation (Table 1). One of the rVSV-wt animals (362-09) did not show any discernable neurological signs. No neurological deficits were observed in any of the macaques in the vehicle control, rVSV-ZEBOV-GP, or rVSV-MARV-GP groups. Hematology and serum biochemistry were also monitored for each animal with no changes being observed in any animal, when compared to a prebleed for each animal, over the course of the study (data not shown).

**Table 1:** Daily and group mean neurologic scores of animals inoculated IT.

Group	Animal #	Clinical Scores	
		Daily Mean	Group Mean
Vehicle Control	53-09	0	0
	60-09	0	
	68-01	0	
	359-09	0	
rVSV-wt	56-09*	2.8	2
	67-01**	3.2	
	362-09	0	
rVSV-ZEBOV-GP	352-09	0	0
	354-09	0	
	357-09	0	
	358-09	0	
	360-09	0	
	361-09	0	
	363-09	0	
rVSV-MARV-GP	52-09	0	0
	57-09	0	
	59-09	0	
	61-09	0	
	355-09	0	
	356-09	0	
	364-09	0	

0 = No clinical signs of encephalitis; 1 = Rough coat, not eating; 2 = High pitch vocalization, inactive, slow moving; 3 = Shaky movements, tremors, incoordination, limb weakness; 4 = Inability to stand, limb paralysis, moribund.

*: 56-09 was euthanized at day 6 post inoculation with a clinical score of 3. The animal was assigned a clinical score of 4 for the remainder of the 21 day experimental period.

**: 67-01 was euthanized at day 5 post inoculation with a clinical score of 3. The animal was assigned a clinical score of 4 for the remainder of the 21 day experimental period.

### Detection of rVSV and rVSV RNA in swabs, blood, and tissues

Swabs and blood collected over the 21 day study (Fig. 1B), and tissues collected at necropsy were prepared for virus and RNA isolation to detect rVSV by plaque assay and rVSV genomic and antigenomic RNA by TaqMan quantitative real-time (qRT)-PCR as an indicator of rVSV replication. The two rVSV-wt macaques that showed clinical signs of neurologic disease (Table 1) also had rVSV and rVSV genome in the frontal cortex sections with one animal (56-09) having both rVSV and rVSV genome in the left and right hemisphere frontal cortex sections (Table 2). One animal from the rVSV-ZEBOV-GP group (358-09) had low levels of rVSV RNA detectable at day 21 post inoculation but no virus was isolated from the tissues (Table 2), while an animal from the rVSV-MARV-GP group (61-09) had low levels of detectable rVSV RNA in axillary lymph node (LN) taken at day 21 post inoculation with no virus isolated from this tissue (Table 2). None of the other macaques had detectable rVSV RNA in neural tissues, spleen, liver, heart, kidney, axillary LN, or adrenal gland (data not shown for non-neural negative tissues). Three animals from the rVSV-ZEBOV-GP group also had detectable rVSV RNA in nasal (357-09), oral (358-09), and rectal (352-09, 358-09) swabs at different times post inoculation with only 357-09 having virus isolated from the nasal swab at day 2 post inoculation (Table 3). None of the animals had detectable levels of rVSV RNA in any blood samples taken (data not shown).

**Table 2:** Viral loads in neural tissue and LN as measured by qRT-PCR (log10 copies/g)/or virus log10.

Group	Animal #	FC-L	OC-L	FC-R	OC-R	CSC	LN
Vehicle Control	53-09	neg@	neg	neg	neg	neg	neg
	60-09	neg	neg	neg	neg	neg	neg
	68-01	neg	neg	neg	neg	neg	neg
	359-09	neg	neg	neg	neg	neg	neg
rVSV-wt	56-09*	**7.8/4.5**	neg	**7/4.2**	neg	neg	neg
	67-01**	**7.2/4.1**	neg	neg	neg	neg	neg
	362-09	neg	neg	neg	neg	neg	neg
rVSV-ZEBOV-GP	352-09	neg	neg	neg	neg	neg	neg
	354-09	neg	neg	neg	neg	neg	neg
	357-09	neg	neg	neg	neg	neg	neg
	358-09	**4.2/0**	neg	neg	neg	neg	neg
	360-09	neg	neg	neg	neg	neg	neg
	361-09	neg	neg	neg	neg	neg	neg
	363-09	neg	neg	neg	neg	neg	neg
rVSV-MARV-GP	52-09	neg	neg	neg	neg	neg	neg
	57-09	neg	neg	neg	neg	neg	neg
	59-09	neg	neg	neg	neg	neg	neg
	61-09	neg	neg	neg	neg	neg	**5.4/0**
	355-09	neg	neg	neg	neg	neg	neg
	356-09	neg	neg	neg	neg	neg	neg
	364-09	neg	neg	neg	neg	neg	neg

@ Negative (neg) result below the detection of assay at 4 log10 copies/g of tissue. FC-L = left frontal cortex, OC-L = left occipital cortex, FC-R = right occipital cortex, OC-R = right, occipital cortex, CSC = cervical spinal cord, LN = lymph node; rVSV vaccine inoculation occurred in left hemisphere.

*: euthanized on day 6;

**: euthanized on day.

**Table 3:** Virus and Virus anti/genomes in animal swabs as measured by virus isolation and qRT-PCR.

		Day/Animal #/Virus isolation log_10_		
Group	No. Positive	Nasal	Oral	Rectal
Vehicle Control	0^A^	neg@	neg	neg
rVSV-wt	0^B^	neg	neg	neg
rVSV-ZEBOV-GP	3^C^			7+++/352-09/0
		2++/357-09/3.3		
			21/358-09/0	14++/358-09/0
rVSV-MARV-GP	0^D^	neg	neg	Neg

@ Negative (neg) result below the detection of assay at 4 log10 copies/g of tissue. 21 = >4 log10 copies/swab,

**++:**  = 5 log_10_ copies/swab,

**+++:**  = >6 log_10_ copies/swab.

### Histopathological examination of brain and spinal cord

**Figure 2:**
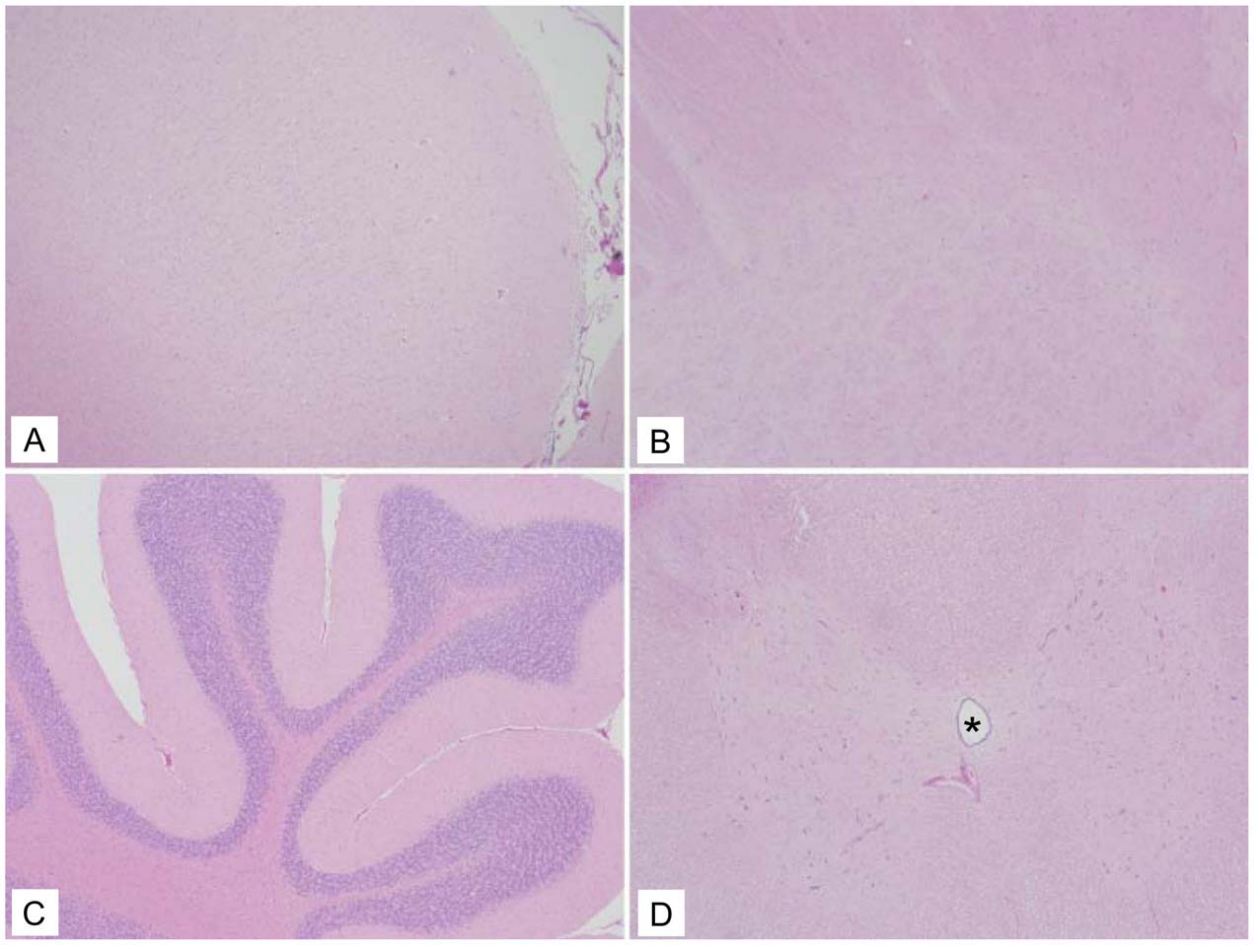
Representative vehicle control histology with no lesions in the neural tissue. (A) Frontal cortex (10×). (B) Basal ganglia (10×). (C) Cerebellum (10×). (D) Spinal Cord (Asterisk denotes central canal) (10×).

The four macaques that received the vehicle control IT in both hemispheres had no inflammatory lesions in any of the brain or spinal cord sections (Fig. 2, Table 4) examined except for one section from one animal (359-09), which had a single meningeal and parenchymal perivascular cuff of lymphocytes in the right thalamus near the vehicle control inoculation site (Table 4).

**Table 4:** Histopathological scores from left/right hemisphere of the brain and spinal cord.

Group	Animal #	Day PI	FC	BG	TH	OC	CB/BS	SC
Vehicle Control	53-09	21	0/0	0/0	0/0	0/0	0/0	0
	60-09	21	0/0	0/0	0/0	0/0	0/0	0
	68-01	21	0/0	0/0	0/0	0/0	0/0	0
	359-09	21	0/0	0/0	0/0	0/0	0/0	0
rVSV-wt	56-09*	6	4/4	4/4	3/2	1/1	2/2	3
	67-01**	5	2/3	2/1	0/0	0/0	0/1	1
	362-09	21	4/1	1/1	1/1	0/0	0/2	3
rVSV-ZEBOV-GP	352-09	21	1/1	1/0	1/0	0/0	0/0	0
	354-09	21	1/1	0/0	1/0	0/0	0/0	0
	357-09	21	1/0	0/0	0/0	0/0	1/0	0
	358-09	21	0/0	1/0	1/0	0/1	0/0	0
	360-09	21	1/0	0/0	0/0	0/0	0/0	0
	361-09	21	1/0	0/0	0/0	0/0	0/0	0
	363-09	21	0/0	0/0	0/0	0/0	0/0	0
rVSV-MARV-GP	52-09	21	0/1	1/0	1/0	0/0	0/0	0
	57-09	21	0/0	0/0	0/0	0/0	0/0	0
	59-09	21	1/0	0/0	1/0	0/0	0/0	0
	61-09	21	0/0	0/0	0/0	0/0	0/0	0
	355-09	21	1/0	1/4	0/0	0/0	0/0	0
	356-09	21	1/1	0/0	0/0	0/0	0/0	0
	364-09	21	1/0	0/0	0/1	0/0	0/1	0

Left score #/Right score #; Scores; 0 = no lesions, 1 = minimal perivascular inflammatory infiltrate, no gliosis, no neurodegeneration, no satellitosis, and no necrosis, 2 = mild perivascular inflammatory infiltrate, mild gliosis, no neurodegeneration, no satellitosis, and no necrosis, 3 = moderate inflammatory infiltrate with moderate gliosis, mild neurodegeneration, mild satellitosis, and mild necrosis, and 4 = severe inflammatory infiltrate with moderate to severe gliosis, neurodegeneration, satellitosis, and necrosis.

FC = frontal cortex, BG = basal ganglia, TH = thalamus, OC = occipital cortex, CB/BS = cerebellum/brain stem, SC = spinal cord; PI = post-inoculation.

**Figure 3:**
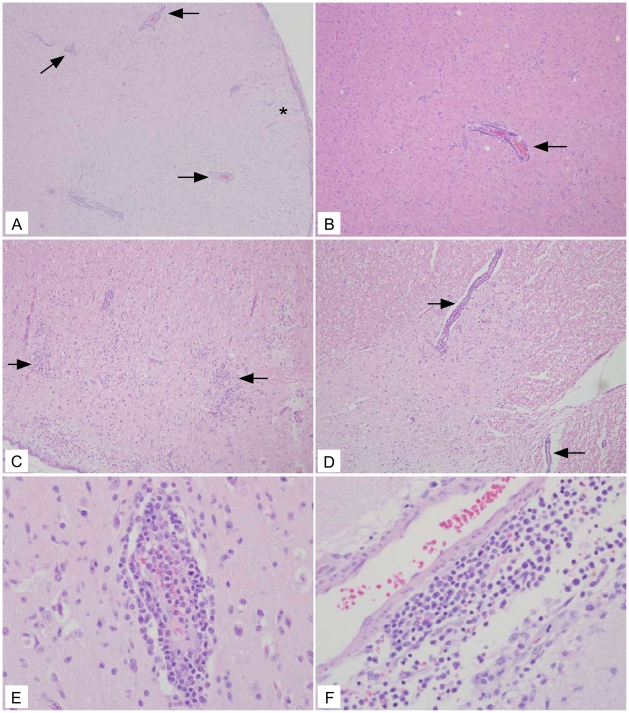
Representative rVSV-wt histology showing lesions in all neural tissue examined. (A) Frontal cortex (10×) section with severe encephalitic changes including perivascular lymphohistocytic cuffs (arrows) and aggregates of lymphocytes in the neuroparenchyma (*). (B) Frontal cortex (10×) section with perivascular cuff of lymphocytes and histocytes (arrow). (C) Cerebellum (10×) section with aggregates of lymphocytes in the parenchyma (arrows) admixed with increased numbers of reactive glial cells. (D) Spinal cord (10×) section with gliosis admixed with regions of perivascular inflammation (arrows). (E) Frontal cortex (40×) section depicting large numbers of perivascular lymphocytes and histocytes infiltrating into the adjacent gray matter. (F) Basal ganglia (40×) section depicting large numbers of lymphocytes and histocytes both around a meningeal vessel and invading into the adjacent tissue.

The three macaques that received rVSV-wt had the most profound histologic lesions (Table 4). Lesions were most pronounced in the cortices and the basal ganglia, with lesions of lesser severity encountered in the caudal brainstem and cerebellum. Lesions consisted of marked perivascular cuffs and parenchymal aggregates of lymphocytes, macrophages, and fewer plasma cells (Fig. 3, arrows and panels E and F). Lymphocytes often replaced lost neurons and there was scattered neuronal satellitosis, necrosis, and degeneration. Gliosis was marked in the regions of intense inflammation and the overlying meninges typically contained small to moderate numbers of lymphocytes and macrophages (Fig. 3, panel F). Spinal cord sections from these three animals displayed similar lesions; however, the lesions in one animal (67-01) were milder with only minimal inflammation and scattered neuronal satellitosis (Table 4).

**Figure 4:**
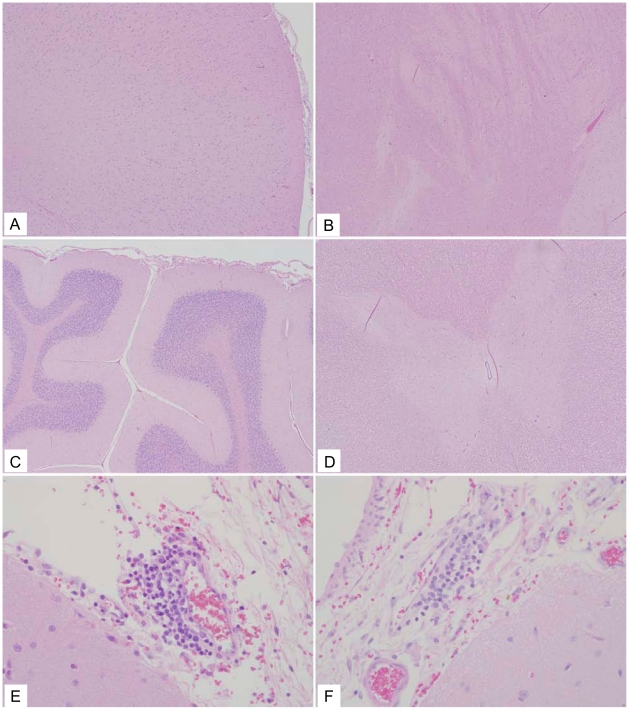
Representative rVSV-ZEBOV-GP histology. (A and B) Frontal cortex (10×) sections with no lesions. (C) Cerebellum (10×) section with no lesions. (D) Spinal cord (10×) section with no lesions. (E) Frontal cortex (40×) section with a mild perivascular cuff of lymphocytes. (F) Occipital cortex (40×) section with a mild perivascular cuff of lymphocytes.

**Figure 5:**
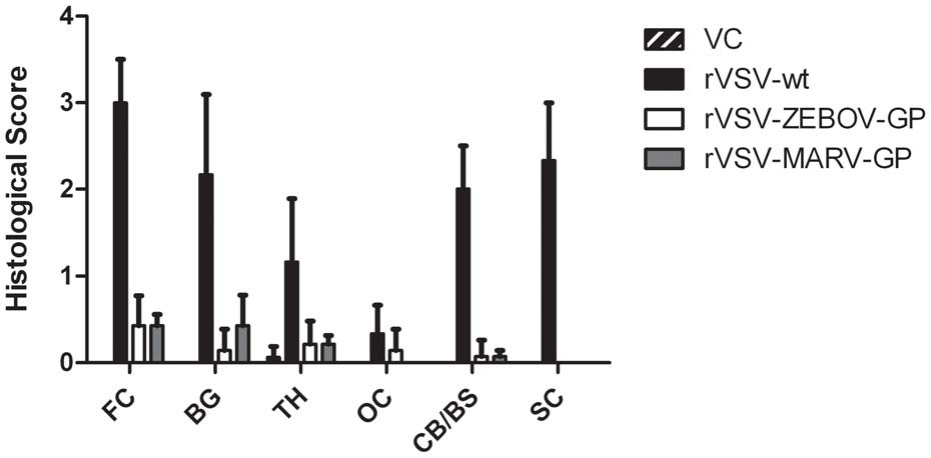
Combined histological scores of neural tissue. Graph displaying the mean histological values of the neural tissue from left and right hemisphere for the frontal cortex (FC), basal ganglia (BG), thalamus (TH), occipital cortex (OC), and cerebellum/brainstem (CB/BS), plus scores from the spinal cord (SC). Error bars, standard deviation. VC = vehicle control.

The seven animals that received rVSV-ZEBOV-GP had a variety of mild histologic changes. One animal (363-09) lacked parenchymal lesions in the brain and spinal cord (Table 4), while 6 of the 7 animals (352-09, 354-09, 357-09, 358-09, 360-09, 361-09) had rare, focal areas of meningeal inflammation in various cerebral sections (Fig. 4, Table 4). Only one animal (357-09) had a small focal area of meningeal inflammation in the cerebellum. There were no spinal cord lesions in any of the rVSV-ZEBOV-GP animals examined (Fig. 4 and 5, Table 4).

**Figure 6:**
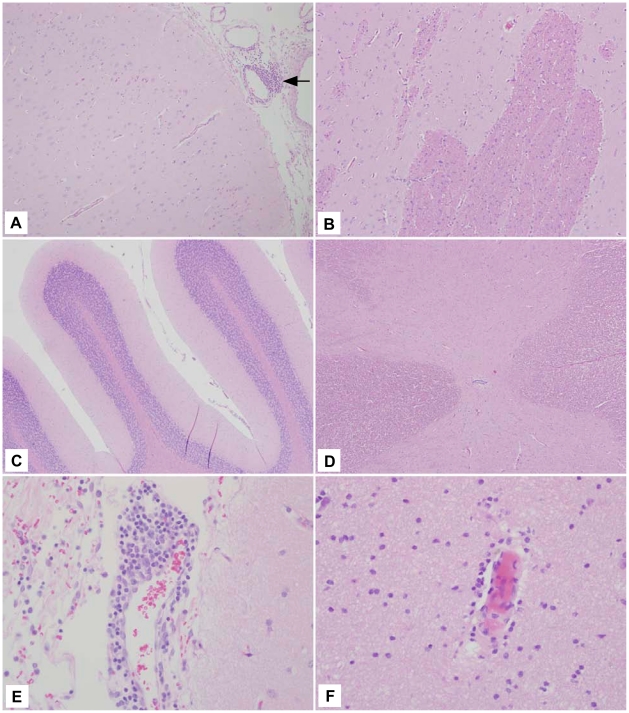
Representative rVSV-MARV-GP histology. (A) Frontal cortex (10×) section from 59-09 that had a small perivascular cuff of lymphocytes. (B) Frontal cortex (10×) section with no lesions. (C) Cerebellum (10×) section with no lesions. (D) Spinal cord (10×) section with no lesions. (E) Frontal cortex (40×) section with a mild perivascular cuff of lymphocytes. (F) Frontal cortex (40×) section with a scant perivascular cuff of lymphocytes.

The macaques that received the rVSV-MARV-GP IT inoculation had varied histology. Two animals (57-09, 61-09) did not have any histologic lesions present in the brain and spinal cord sections examined (Table 4). Two different animals (52-09, 59-09) had mild lesions in the cortex consisting of small perivascular cuffs of lymphocytes and rare macrophages (Table 4). Another animal (364-09) had similar perivascular infiltrates also present in the brainstem (Table 4). Multifocal meningeal, perivascular aggregates of lymphocytes were also seen in two animals (356-09, 364-09) (Fig. 6). Lastly, one animal (355-09) had a focal tract of necrosis in the caudal basal ganglia/proximal thalamus that corresponded to the injection site. No brain lesions were observed away from the injection site in any of the rVSV-MARV-GP IT animals (Table 4).

Figure 5 represents the average of the combined left and right hemisphere scores from Table 4 for each group in the study in order to compare the combined histological scores from neural tissues. Overall, comparisons between the rVSV-wt group and the rVSV-ZEBOV-GP or rVSV-MARV-GP groups show that the difference between scores were significant (p = 0.0016 and p = 0.0019 respectively). From these data the rVSV filovirus GP vaccines appear to have no substantial NV in cynomolgus macaques.

## Discussion

More than seven years ago, rVSV vectors expressing foreign GPs from EBOV and MARV were developed and characterized [34]. These rVSV vaccine vectors were subsequently used in cynomolgus and rhesus macaques to assess their ability to protect animals from lethal challenge with ZEBOV, SEBOV, CIEBOV, and MARV, respectively [8], [9], [11], [12], [18], [35]. While the results of these initial experiments were promising, VSV and rVSV have displayed NV in experimental settings using rodents [41], [42], [43], [44], [45], [46], [47], [48], [62] and in cynomolgus and rhesus macaques that were inoculated intracerebrally [49], [59]. The NV seen in these experimental settings raised the question about the safety and NV of the replication-competent rVSV filovirus GP vaccines. To address this question we compared vehicle control, rVSV-wt, rVSV-ZEBOV-GP, and rVSV-MARV-GP since these vaccine vectors are replication-competent. We modeled our NV test on the platform and scoring system employed for the yellow fever virus vaccine [55] and rVSV HIV vaccine vectors [59].

Contrary to the rVSV-wt cohort, none of the macaques in either the vehicle control group or the two filovirus vaccine groups displayed clinical neurological abnormalities (Table 1). This pattern was also seen when comparing the amount of detectable rVSV RNA in the macaques as two rVSV-wt animals (56-09, 67-01) had the most significant amount of rVSV and rVSV RNA detected in the neural tissue (Table 2). In particular, it was interesting that one of these animals (56-09) also had rVSV and rVSV RNA detectable in the right frontal cortex, considering that the experimental inoculation was in the left hemisphere thalamus suggesting spread of rVSV-wt through the neural tissues. Histopathologic analysis confirmed that the rVSV-wt virus is NV in cynomolgus macaques causing severe encephalitis when inoculated IT. All three animals in this cohort displayed varying degrees of inflammation and neurodegeneration in the brain and spinal cord sections evaluated with one of the macaques (56-09) having the most severe scores throughout the neural tissue (Table 4) which correlated with the detectable amounts of rVSV RNA in neural tissue (Table 2). None of the animals in the rVSV filovirus GP vaccine or vehicle control groups showed any evidence of neurodegeneration or neuroinflammation. Animals in both rVSV filovirus GP vaccine groups had occasional perivascular lymphocytes and meningeal infiltrates primarily in the frontal cortex and thalamus (Fig. 6, Table 4); however, there was no spread into the adjacent parenchyma and no evidence of neurodegeneration, satellitosis, or gliosis. It is also interesting to note that there were no lesions seen in the spinal cord for the two rVSV filovirus GP vaccine groups whereas there were lesions seen in the rVSV HIV vaccine IT-inoculated macaques in a previous study [59]. The difference between the lesions seen for rVSV HIV vaccine IT-inoculated macaques and our study could be the fact that the HIV vaccines still contain the VSV G protein whereas the rVSV filovirus GP vaccines lack the VSV G protein. Also, as described by Johnson et al., this observation could have resulted from contamination of the cerebrospinal fluid while performing the IT inoculation. This could be the case, although we observed lesions in the spinal cord for all macaques in the rVSV-wt group including one animal (362-09) which showed no major clinical neurologic disease symptoms and had no detectable rVSV RNA at day 21 in the neural tissues. Taken together, the histological lesion scores confirm that neither rVSV-ZEBOV-GP nor rVSV-MARV-GP is significantly NV and neither causes significant neuropathology in the cynomolgus macaque when inoculated IT as compared to clarified Vero cell culture fluid and rVSV-wt. It is interesting to note that the very mild inflammation seen in some of the vaccine cohorts in our study are far below that which is deemed acceptable for the Mumps vaccine in many countries as recently reviewed [63]. Although the neurotropic differences of viruses are difficult to compare our findings that the rVSV filovirus GP vaccines are similar to and even further attenuated for NV than a widely used vaccine are encouraging.

Recovery of detectable rVSV RNA from swabs seemed to correlate with small lesions seen in some of the rVSV-ZEBOV-GP animals (357-09, small lesion in meninges of cerebellum 358-09, small lesion in meninges of right basal ganglia), whereas one animal (352-09) had no detectable lesions (Table 4). Low amounts of rVSV RNA were detected in the left frontal cortex of one animal (358-09), despite the detection of rVSV RNA at this site there were no spinal cord lesions in this animal or in any other neural tissue sampled from this animal. These observations combined with the swab data for this animal suggests the rVSV-ZEBOV-GP virus may have moved beyond the neural tissue from the site of inoculation. This thought is interesting considering EBOV and MARV initially target dendritic cells and macrophages *in vivo* [64], [65]. The fact that these rVSV vectors express filovirus GP and have the protein on the virion surface, which would target the vectors to dendritic cells and macrophages, could explain why in a few cases they were detected in areas outside of the neural tissue perhaps targeting dendritic cells and macrophages that appeared at the IT inoculation sites which then migrated to LN (Table 2) or other sites (Table 3) where the RNA was able to be isolated. In fact, only the day 2 nasal swab from 357-09 was positive for virus isolation (Table 3) with low levels of virus. Considering the lack of shedding of these vaccines in the previous studies this was interesting. There are a few possibilities as to why virus may have been detected in this study: transfer of virus along neurons through the olfactory bulb to the olfactory neurons, or the fact that the nasal swab procedure sometimes results in minor bleeding regardless of how gentle the nares are swabbed, which could have picked up a low level viremia not picked up by qRT-PCR.

The lack of NV in the rVSV filovirus GP vaccine vectors when compared to rVSV-wt is encouraging considering that the vaccines are not attenuated through mutation of known virulence markers such as methionine 51 of the VSV M protein [66], [67]. These results are similar to studies with VSV vectors expressing influenza hemagglutinin (HA) protein which are completely attenuated for pathogenesis in the mouse model [24] when compared to rVSV-wt. These study results should perhaps not be surprising considering the glycoprotein(s) of a virus generally contributes to cell tropism, and when compared to VSV, the filoviruses and influenza virus do not show a neural tropism. The deletion of VSV G and replacement of this protein with glycoproteins from foreign viruses that do not have a neural tropism would suggest the rVSV-(foreign glycoprotein) would not be NV. However, even though glycoproteins contribute to cell tropism other viral factors that also contribute cannot be ruled out, which is why NV testing is important when evaluating vaccine safety. The clinical, histological, and virus replication data from this study indicate that the rVSV-ZEBOV-GP and rVSV-MARV-GP vectors lack the NV properties of the rVSV-wt vector.

In addition to this NV study, the rVSV-ZEBOV-GP vector has been safety tested in two animal models with defective immune systems, NOD-SCID mice [68] and SHIV-infected rhesus monkeys [35]. Upon immunization, no evidence of overt illness was noted in any of these immunocompromised animals. Also, the rVSV-ZEBOV-GP vector was recently used to treat a laboratory worker after a recent laboratory accident without any major complications [40]. We have previously shown that rVSV filovirus GP vectors have efficacy as preventive vaccines and postexposure treatments and have promising safety profiles. These data together with findings from our current NV study suggest that the rVSV filovirus GP vaccine system is ready for advanced development for human use.
